# The effects of home-based exercise therapy for breast cancer-related fatigue induced by radical radiotherapy

**DOI:** 10.1007/s12282-022-01408-3

**Published:** 2022-10-14

**Authors:** Georgios Mavropalias, Prue Cormie, Carolyn J. Peddle-McIntyre, Daniel A. Galvão, Dennis R. Taaffe, Christelle Schofield, Sharon Ray, Yvonne Zissiadis, Robert U. Newton

**Affiliations:** 1 Exercise Medicine Research Institute, Edith Cowan University, 270 Joondalup Drive, Joondalup, WA 6027 Australia; 2 School of Medical and Health Sciences, Edith Cowan University, Joondalup, Australia; 3 Centre for Molecular Medicine and Innovative Therapeutics, and Centre for Healthy Aging, Health Futures Institute, Murdoch University, Perth, Australia; 4 Discipline of Exercise Science, Murdoch University, Perth, Australia; 5 Peter MacCallum Cancer Centre, Melbourne, Australia; 6 Department of Radiation Oncology, Genesis Cancer Care, Perth, Australia; 7 School of Human Movement and Nutrition Sciences, University of Queensland, Brisbane, Australia

**Keywords:** Radiotherapy, Breast cancer, Quality of life, Behavior change, Home-based exercise, Cancer-related fatigue

## Abstract

**Background:**

Radiotherapy (RT) can lead to cancer-related fatigue (CRF) and decreased health-related quality of life (HRQoL) in breast cancer patients. The purpose of this trial was to examine the feasibility and efficacy of a home-based resistance and aerobic exercise intervention for reducing CRF and improving HRQoL in breast cancer patients during RT.

**Methods:**

Women with breast cancer (*N* = 106) commencing RT were randomized to 12 weeks of home-based resistance and aerobic exercise (EX) or usual care/control (CON). The primary endpoint was CRF, with secondary endpoints of HRQoL, sleep duration and quality, and physical activity. Measurements were undertaken prior to RT, at completion of RT (~ 6 weeks), at completion of the intervention (12 weeks), and 6 and 12 months after RT completion, while CRF was also measured weekly during RT.

**Results:**

Eighty-nine women completed the study (EX = 43, CON = 46). Over the 12-week intervention, EX completed 1–2 resistance training sessions and accumulated 30–40 min of aerobic exercise weekly. For CRF, EX had a quicker recovery both during and post-RT compared to CON (*p* < 0.05). Moreover, there was a significant difference in HRQoL between groups at RT completion, with HRQoL unchanged in CON and higher in EX (*p* < 0.05). There was no change in sleep duration or quality for either group and there were no exercise-related adverse effects.

**Conclusions:**

Home-based resistance and aerobic exercise during RT is safe, feasible, and effective in accelerating CRF recovery and improving HRQoL. Improvements in CRF and HRQoL for these patients can be achieved with smaller exercise dosages than stated in the generic recommendations for breast cancer.

## Introduction

Breast cancer (BCa) is the most common form of cancer among women. In Australia, 1 in 8 women will be diagnosed with BCa by the age of 85 [1]. Radiotherapy (RT) treatment is an important component of breast cancer treatment and is used with curative intent as well as for palliation. One commonly reported adverse side effect of RT is cancer-related fatigue (CRF) and is estimated to affect between 70 and 100% of patients [2]. The persistent tiredness associated with CRF is experienced during and after treatment and has a substantial negative impact on health-related quality of life (HRQoL), significantly interfering with daily function and can compromise the ability to complete treatment, especially in females compared to males in all types of cancer [3]. Although the precise mechanisms associated with CRF have yet to be identified, the driving factors are commonly theorized to be associated with negative physiological (i.e., muscle strength and endurance, cardiorespiratory fitness, body composition), biologic/hematologic (i.e., inflammatory response, metabolic/endocrine/immune function), psychological (i.e., anxiety, depression, distress), behavioral (i.e., sleep quality and quantity, appetite) and social (i.e., social interaction) changes resulting from cancer and its treatment [3, 4]. Historically, patients have been advised to rest during and after cancer treatments; however, research evidence refutes the use of rest as an effective strategy to manage CRF due to the detrimental effects of inactivity on structure and function (i.e., negative adaptations in the neuromuscular, skeletal and cardiorespiratory systems) [5].

Exercise could offer a potent stimulus to counteract CRF as it elicits positive adaptations in most of the factors believed to be associated with CRF, HRQoL, and sleep [6, 7]. However, while exercise has been shown to not increase CRF, very little research has examined the use of exercise as a management strategy for CRF during RT in women with BCa, as the majority of research conducted during treatment has involved patients on chemotherapy [8]. In other types of cancer, investigations of aerobic exercise during radical external beam RT have revealed that walking programs were effective in mitigating CRF in prostate cancer patients [9, 10]. Similar to results observed following chemotherapy, significant increases in CRF were observed in control patients but not in exercising patients throughout treatment [9, 10]. Due to the prevalence of RT in BCa care, and the severity of CRF during RT especially in women [3], there is an urgent need for more research on the effects of different forms of exercise on CRF during and after RT in women with BCa.

It has previously been shown that higher resistance and aerobic exercise intensity can significantly reduce CRF and improve sleep in patients undergoing chemotherapy for BCa [7, 11]. Moreover, we have previously shown that exercise dosage (i.e., repetitions, intensity, duration) can significantly affect health and fitness outcomes in men with prostate cancer [12], therefore it is necessary to record exercise dosage to not only ensure compliance with the intervention, but to also investigate if and how CRF can influence the ability to follow the exercise prescribed. One pilot trial has demonstrated that a 4-week home-based walking and resistance exercise program was effective in reducing CRF in women with BCa beginning RT [13]. However, this was a mixed patient group with BCa and prostate cancer, very short duration intervention, and with only a 3-month follow-up.

Given the above, the purpose of this study was to (a) examine the effects of a 12-week home-based resistance and aerobic exercise program on CRF, HRQoL, and sleep quality and duration in BCa patients during and up to 12 months after RT, and (b) investigate how CRF, HRQoL, and sleep quality and duration affect the participants’ ability to follow their prescribed exercise program.

## Materials and methods

**Fig. 1:**
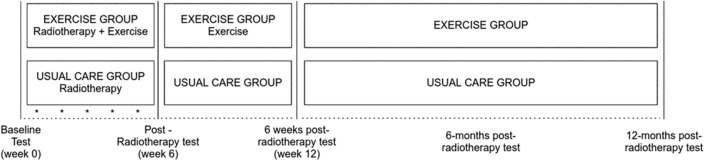
Schematic of the research project design. *The serial assessment of CRF and physical activity level at the start of each week

This was a two-arm, randomized controlled clinical trial (Fig. 1). Participants meeting the inclusion criteria were randomized to either exercise or usual care/control. The exercise group participated in a 12-week home-based exercise program involving resistance and aerobic exercise supplemented by a 1-h face-to-face consultation, four 30-min telephone consultations, an exercise manual, and exercise equipment. Assessments were conducted prior to initiating radiotherapy (week 0), at completion of RT (~ 6 weeks), at completion of the intervention (12 weeks), and 6 and 12 months after completion of RT, and consisted of CRF, HRQoL, sleep duration and quality, and physical activity. Radiotherapy completion rates, and adverse effects of RT and exercise, were assessed each week during the intervention. All participants had completed a written informed consent and medical questionnaire approved by the institution’s human research ethics committee before participating in the study (ANZCTR registration number: ACTRN12611001266954).

### Participants

**Fig. 2:**
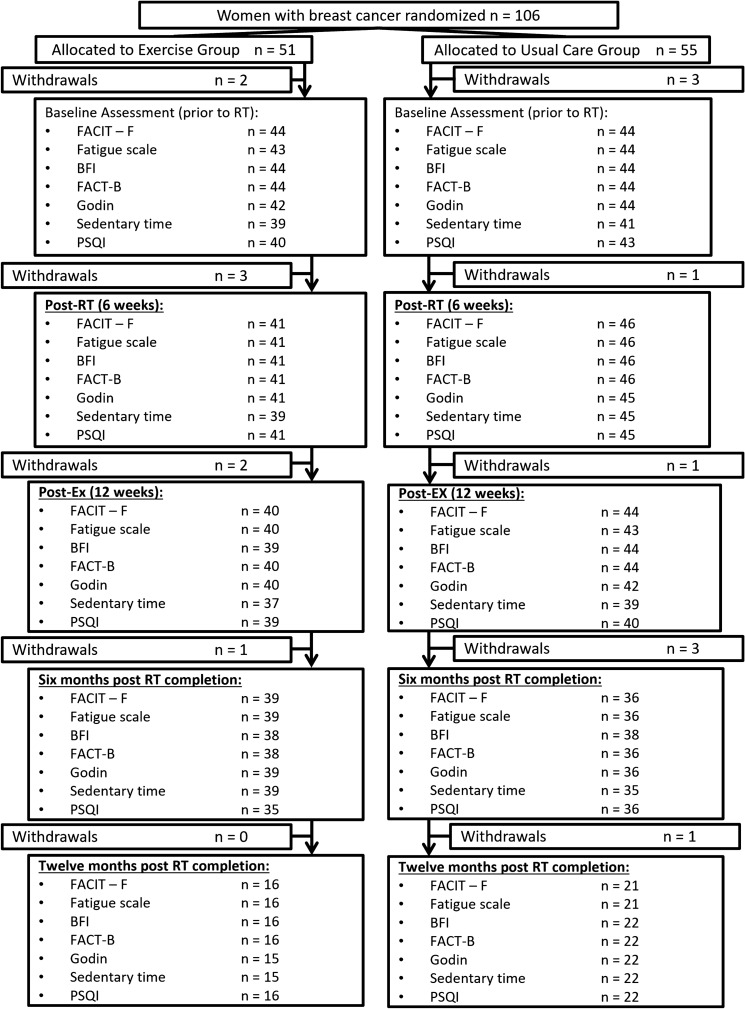
Consort diagram of the study

One hundred and six (*N* = 106) women with stage I-III BCa scheduled to receive radical RT were randomized (Fig. 2) into exercise (*N* = 51) or usual care (*N* = 55). Inclusion criteria consisted of a histological diagnosis of BCa and prescribed radical RT treatment for 6 weeks. Exclusion criteria consisted of (a) bone metastatic disease; (b) any cardiovascular, musculoskeletal, or neurological condition that could inhibit them from exercising or put them at risk during exercise; and (c) difficulties reading and/or understanding English. Balanced randomization procedures were utilized to allocate participants into the two study arms at a ratio of 1:1 stratified for treatment history (chemotherapy vs. no chemotherapy; hormone therapy vs. no hormone therapy) and age (≤ 55 vs. > 55). Randomization was conducted by a researcher who had no contact with participants.

### Measurements

Assessment of primary and secondary outcome measures took place at: (1) baseline (i.e., week 0, prior to initiating RT and the intervention period); (2) post-RT (i.e., 6 weeks after baseline, after completing RT and mid-way through the intervention period); (3) post-exercise (i.e., 12 weeks after baseline); and (4) follow-up (i.e., 6 and 12 months after the completion of RT). Additionally, serial assessments of CRF (FACIT-F), physical activity, and exercise dosage (through a logbook completed by the participants) were conducted each week throughout RT. All assessments were conducted during scheduled visits to the radiation oncology clinic.

### Primary endpoints

Cancer-related fatigue was assessed using the Functional Assessment of Chronic Illness Therapy-Fatigue (FACIT-F) questionnaire. The FACIT-F is a 13-item scale commonly used to assess fatigue in cancer patients [14], including those receiving exercise interventions [15]. Validity and reliability of the measurement tool has been established, and a recent systematic review of all tools used to measure CRF recommended the use of the FACIT-F [16, 17]. A score of ≤ 34 in the FACIT-F has been proposed as a cut-off point for clinically meaningful CRF, while a change of ≥ 4 has been established as a clinically significant change in CRF [14, 18]. Lower scores in the FACIT-F indicate greater CRF.

Fatigue was also assessed using the Brief Fatigue Inventory (BFI) at baseline, post-RT, post-exercise, and at follow-up. The BFI is a reliable instrument that allows for the rapid assessment of fatigue level in cancer patients [19]. It consists of nine items for quantifying fatigue in the past that are rated on a 0 – 10 scale where 0 is no fatigue or does not interfere and 10 is bad fatigue or completely interferes with activity/work. Higher scores in the BFI indicate greater CRF.

### Secondary outcome measures

#### Quality of life

HRQoL was assessed using the Functional Assessment of Cancer Therapy for patients with BCa (FACT-B + 4) [20], to assess a variety of domains including physical wellbeing, social/family wellbeing, emotional wellbeing, functional wellbeing, and BCa specific domain. FACT-B is an integrated instrument to assess HRQoL in BCa patients and has been extensively employed in clinical trials [21]. Moreover, a BCa symptom-specific four-item arm scale was developed to supplement the FACT-B, forming the FACT-B + 4 [21]. Higher scores in the FACT-B + 4 indicate greater HRQoL.

#### Sleep duration and quality

Insomnia, poor sleep quality and short sleep durations are the most common problems seen in cancer patients [7]. To assess sleep quality and duration, the Pittsburgh Sleep Quality Index (PSQI) questionnaire was used [22]. This tool has been previously used to evaluate sleep disorders in cancer patients [22]. Seven sleep components are assessed in the PSQI which includes subjective sleep quality, latency, duration, efficiency, disturbances, medication use, and daytime dysfunction [23]. These components are rated on a 0–3 scale with lower scores indicating better sleep quality. The components are summed to obtain a global sleep quality score ranging from 0 to 21, with scores > 5 indicative of poor sleep quality [22].

#### Physical activity

Physical activity was assessed using the Godin Leisure-Time Exercise Questionnaire [24] to assess the mean frequency and duration of mild, moderate, and strenuous exercise in a typical week in the past month. Moderate and vigorous physical activity (MVPA) was calculated by combining minutes with a double weighting on vigorous intensity minutes [25]. Participants in the exercise group were instructed to only include exercise outside the intervention.

#### Radiotherapy completion rates and adverse side effects

Adherence to prescribed RT treatments was recorded using standard clinical measures. Completion rate was reported as the percentage of the planned dose and planned fractions completed during the treatment course. The presence and severity of any adverse side effects was assessed using the National Cancer Institute Common Terminology Criteria for Adverse Events (CTCAE—Version 5.0) [26].

#### Adherence to and adverse side effects from the exercise program

Adherence to the exercise program was recorded using detailed logbooks. The frequency, duration and intensity of exercise was examined for both aerobic and resistance exercise. The occurrence and severity of any adverse events including musculoskeletal complications (muscle strains, fractures, etc.) were recorded during the 12-week intervention period, and assessed using the CTCAE—Version 5.0 [26].

### Exercise

The 12-week home-based exercise intervention was a combination of resistance and aerobic exercise. Each participant completed the pre-exercise questionnaire and medical history, and then had a 1-h consultation with an accredited exercise physiologist. The resulting exercise program was relative to the level of fitness and CRF each patient presented with, and individualized to their personal preferences and any pre-existing conditions [6, 27, 28]. The exercise prescription was progressive with participants set an ultimate target of meeting the recommended physical activity levels for cancer patients as outlined by national guidelines [29]. This goal equated to moderately intense aerobic exercise 30 min/day 5 days/week (or vigorously intense aerobic exercise 20 min/day for 3 days/week) and 8–10 strength-training exercises, 8–12 repetitions per exercise for 2–3 days/week. The intervention involved a combination of education and self-management strategies implemented through a variety of methods to achieve this target. Specifically, the intervention involved (1) recommendation and regular reinforcement from an oncologist; (2) a 1-h face-to-face consultation with an accredited exercise physiologist to provide hands on exercise instruction, individualize exercise prescription, and assist the participant to develop strategies to overcome personal barriers especially relating to managing CRF and dealing with changes in its severity; (3) 30-min phone consultations with an accredited exercise physiologist every 2 weeks throughout the intervention to monitor individual progress, address any issues reported by the participant, make necessary revisions to the exercise prescription, and field questions; (4) providing exercise equipment including a Gymstick (Gymstick, Finland) (used to perform progressive resistance exercises at home) and a pedometer (Polar, Finland) (used to monitor the volume of aerobic exercise through tracking the number of steps taken each day); and (5) an exercise manual and log book which provided thorough and clear instructions on why exercise is important, what exercises to perform and how to perform them, as well as detailed diaries to track exercise adherence and intensity. Intensity was recorded using Borg’s 6–20 rating of perceived exertion (RPE) scale [30].

Participants randomized to the usual care group received standard usual care throughout the intervention period. The usual care group did not receive any recommendation or support to exercise but were not advised or requested to change their exercise behavior or avoid exercise. At the completion of the follow-up assessment (12 months), this group was provided with the exercise manual and logbook, behavioral guidebook and provided with information of exercise programs available for cancer survivors.

### Statistical analysis

As this was a pragmatic trial, we aimed to recruit as many patients as possible. To calculate the achieved power, we used the primary endpoint of FACIT-F score at immediately post-RT, as we hypothesized that the effect of exercise on CRF would be the strongest at that time point. Achieved power was calculated using G*Power (Version 3.1.9.7, Universitat Kiel, Germany). At post-RT, the difference between groups had an effect size of *d* = 0.48. With an alpha level of 0.05 and a one-tail design, the achieved power was 68%, which allowed us to detect moderate effect sizes [31].

A linear mixed-effects model was used with participant ID as the random-effects factor, while fixed-effects factors consisted of demographic and physical characteristics (age, baseline body fat percentage), physical symptoms and conditions (hypertension, high cholesterol, cardiovascular disease, diabetes, osteoporosis), and cancer-specific treatments before and during the intervention (chemotherapy, hormone therapy, surgery), as well as exercise dosage for the exercise group only (number of resistance and aerobic sessions, number of resistance exercises, duration of aerobic exercise, and RPE during resistance and aerobic exercise). The assumption of normality and homoscedasticity of the residuals was verified by visual quantile–quantile plot inspection of the plots and a Shapiro–Wilk test. In the case of a significant interaction effect, pairwise comparisons were performed between conditions and timepoints, with a Holm's *p* value adjustment. The criterion significance level was set at *p* ≤ 0.05. All statistical testing was performed using R 4.1.1 (R Core Team) using the package lmerTest 3.1–3 [32].

## Results

In total, 17 participants withdrew from the study (exercise = 8, usual care = 9), due to (a) difficulty coping with treatment (*n* = 3), (b) finding the filling of questionnaires and logbook burdensome (*n* = 4), (c) due to personal reasons (*n* = 9), and (d) one patient in the usual care group died due to liver failure caused by liver metastasis.

Eighty-nine (84%) participants completed the study (exercise = 43, usual care = 46). Demographic and clinical characteristics of the study population are shown in Table 1. Participants were aged 32–78 years, predominantly married, non-smokers, and currently employed. Regarding cancer therapy prevalence, 93% of the participants in the exercise and 93% in the usual care had undergone surgery, 60% and 59% had undergone chemotherapy, and 35% and 26% had undergone hormone therapy, respectively.

**Table 1:** Participant (*N* = 89) characteristics per group at baseline and radiotherapy description. Values are presented as mean ± standard deviation

Variables	Exercise (*N* = 43)	Usual care (*N* = 46)
Age (y ± SD)	51 ± 10	53 ± 10
Married (*N* (%))	36 (84%)	31 (67%)
Currently employed (*N* (%))	28 (65%)	32 (74%)
Current smoker (*N* (%))	1 (5%)	1 (5%)
Hypertension (*N* (%))	9 (21%)	16 (35%)
Hypercholesterolemia (*N* (%))	11 (26%)	11 (24%)
Cardiovascular disease (*N* (%))	1 (2%)	3 (7%)
Type 2 diabetes (*N* (%))	1 (2%)	2 (4%)
Osteoporosis (*N* (%))	1 (2%)	2 (4%)
Secondary cancer (*N* (%))	0 (0%)	0 (0%)
Had surgery (*N* (%))	40 (93%)	43 (93%)
Had chemotherapy (*N* (%))	26 (60%)	27 (59%)
Had hormone therapy (*N* (%))	15 (35%)	12 (26%)
Baseline time since diagnosis (days ± SD)	147 ± 62	154 ± 70
Baseline time before radiotherapy (days ± SD)	− 22 ± 59	− 12 ± 10
Radiotherapy duration (days ± SD)	35 ± 4	34 ± 4
Radiotherapy total dose (Gy ± SD)	49 ± 2	49 ± 2
Lump bed boost duration (days ± SD)	6 ± 2	6 ± 1
Lump bed boost total dose (Gy ± SD)	10 ± 0	10 ± 0

Characteristics of the radical RT treatment are presented in Table 1. Immediately following the dose to the whole breast, the women received a lump bed boost which refers to a boost dose to the primary tumor bed. The median RT duration was 35 days for both groups with a median dose of 50 Gy, and for the lump bed boost was 6 days with a median dose of 10 Gy. There were RT-related adverse effects, with 4 participants reporting skin burns, 3 reporting viral infection (cold/flu), one participant reporting diarrhea, and one participant reporting vomiting. All the RT-related adverse effects occurred with participants in the exercise group.

**Fig. 3:**
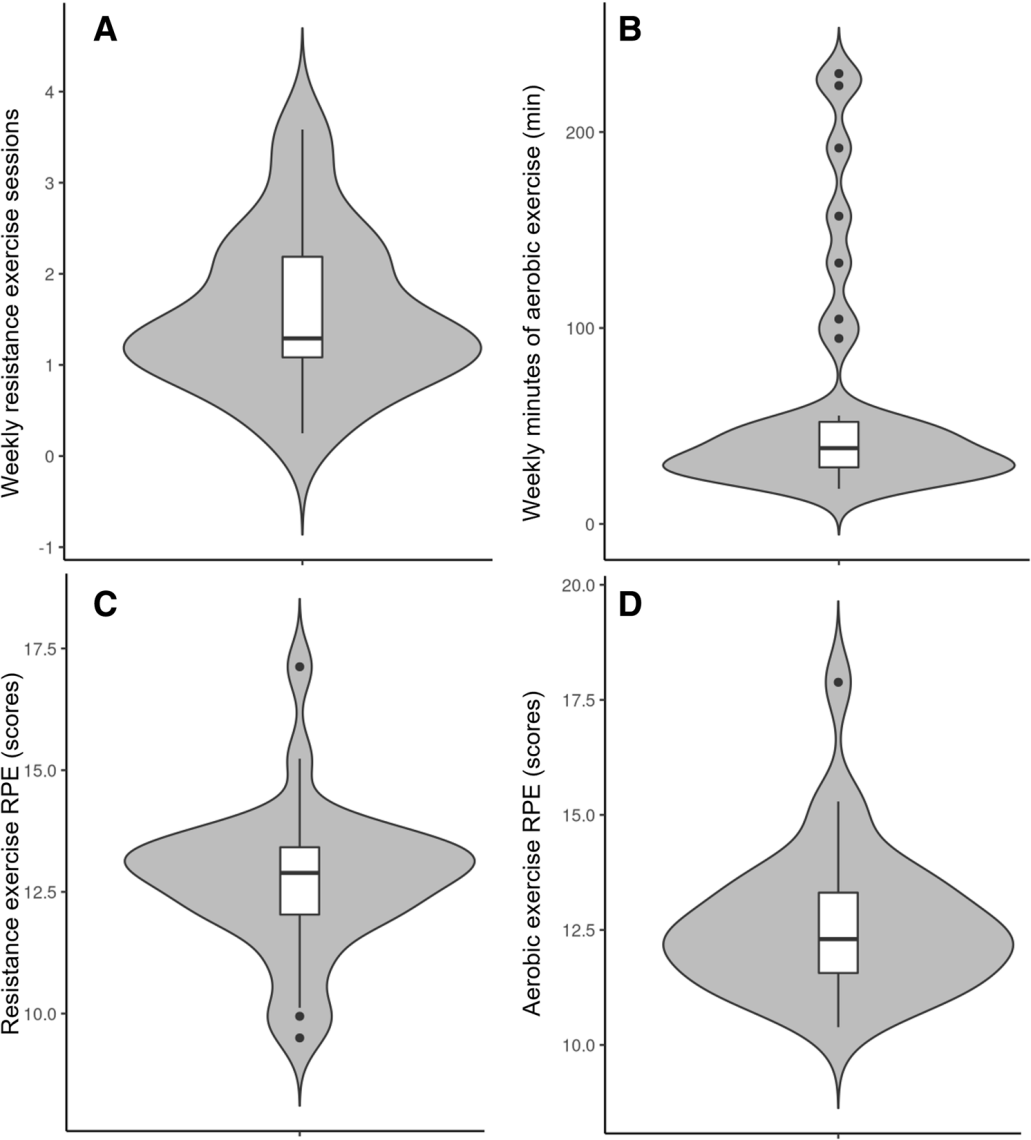
**A** Weekly sessions of resistance exercise, **B** weekly minutes of aerobic exercise, **C** resistance exercise RPE, **D** aerobic exercise RPE. Boxplots indicate median (black line), bottom and top indicate first and third quartiles respectively, and whiskers indicate ± 1.5 IQR

No serious exercise-related adverse events or skeletal fractures were reported during the study; however, one participant rolled her ankle, and 4 reported moderate muscle soreness. Exercise dosage data for the exercise group are presented in Table 2 and Fig. 3. Over the 12-week exercise period, the exercise group completed a median of between 1 and 2 resistance exercise sessions each week. The RPE for the resistance and aerobic exercise sessions ranged between a session median of 12–13. The participants performed 7–8 exercises in each resistance exercise session, while the median weekly duration of aerobic exercise was 30–40 min.

**Table 2:** Exercise dosage

Week	RET session number	RET Exercise number	RET RPE	AET total duration	AET RPE
1	2	8	12	40 (25–68)	13
2	2	8	13	30 (24–68)	13
3	2	8	13	33 (27–61)	13
4	2	8	13	33 (23–63)	12
5	2	8	13	36 (26–59)	12
6	1	8	13	39 (26–52)	13
7	1	8	13	36 (29–66)	12
8	1	8	13	36 (27–62)	12
9	1	8	13	38 (30–54)	12
10	2	8	13	33 (23–57)	12
11	2	8	13	40 (29–52)	13
12	2	8	13	38 (25–61)	12

Median session number, exercise number, and RPE for resistance exercise training (RET), and median session number, total weekly duration (including 1st and 3rd quartiles), and RPE for aerobic exercise training (AET)

### Cancer-related fatigue

**Fig. 4:**
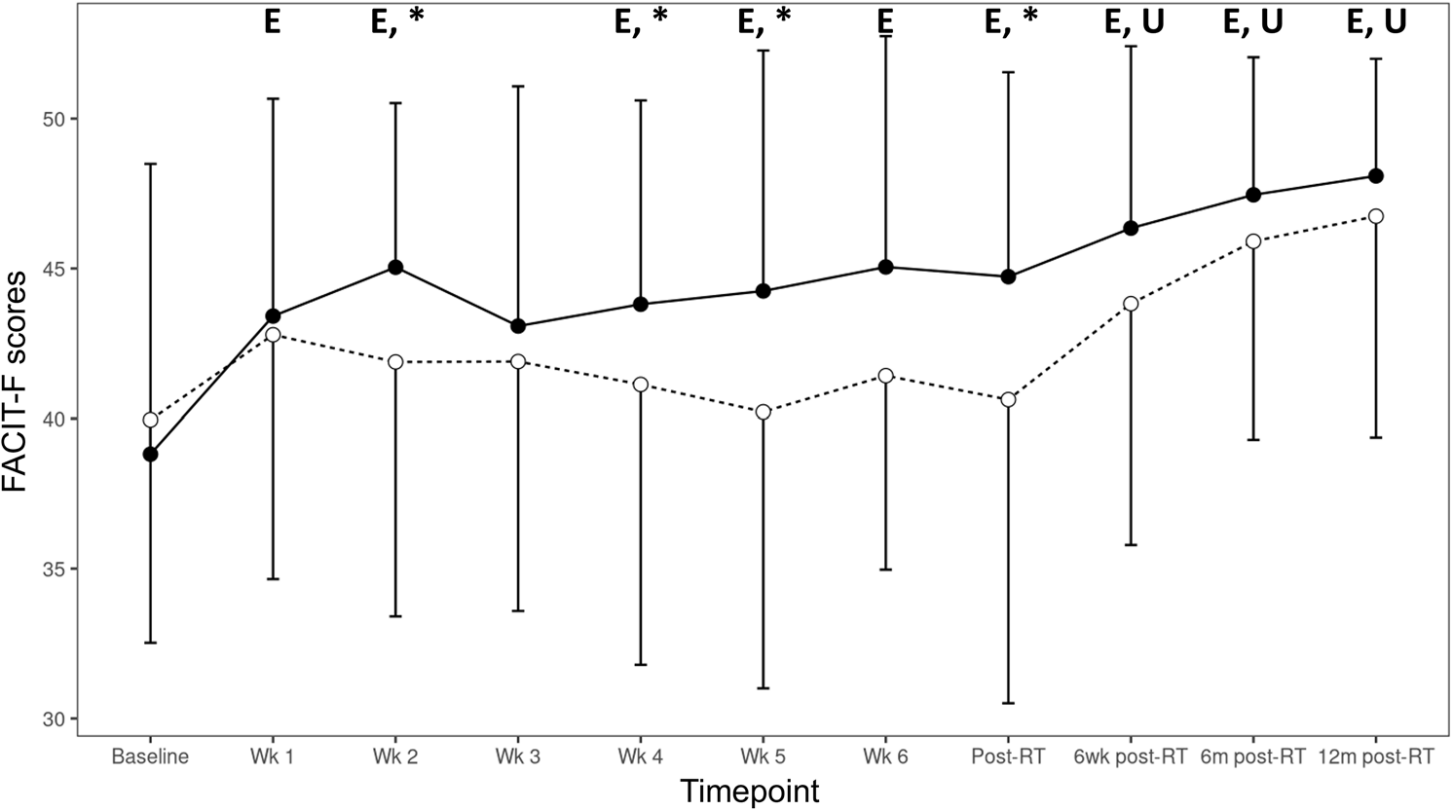
FACIT-F scores for all time points (baseline, weeks 1–6, post-RT, and 6-week, 6-month, and 12-month post-RT) for both groups. Exercise: straight line and black circles; Usual care: dashed line and white circles. Characters denote significant difference of either the exercise (E) or the usual care (U) groups with their respective baselines, and * denotes significant difference between groups (*p* < 0.05). Error bars denote standard deviation of the mean

Based on the FACIT-F fatigue scale, CRF was present at baseline for both groups although not clinically significant [18]. During RT, there was no improvement in the scores of the usual care group, whereas the exercise group had significantly less fatigue compared to baseline in weeks 1, 2, 4, 5 and 6 (Fig. 4). Moreover, there was a significant between-group difference at weeks 2, 4, 5 and post-RT. A significant reduction in fatigue in the usual care group occurred only from 6 weeks post-RT onwards. Immediately post- and at 6 weeks post-RT, being more fatigued was associated with a greater RPE during resistance exercise (*p* = 0.015, *p* = 0.004).

**Fig. 5:**
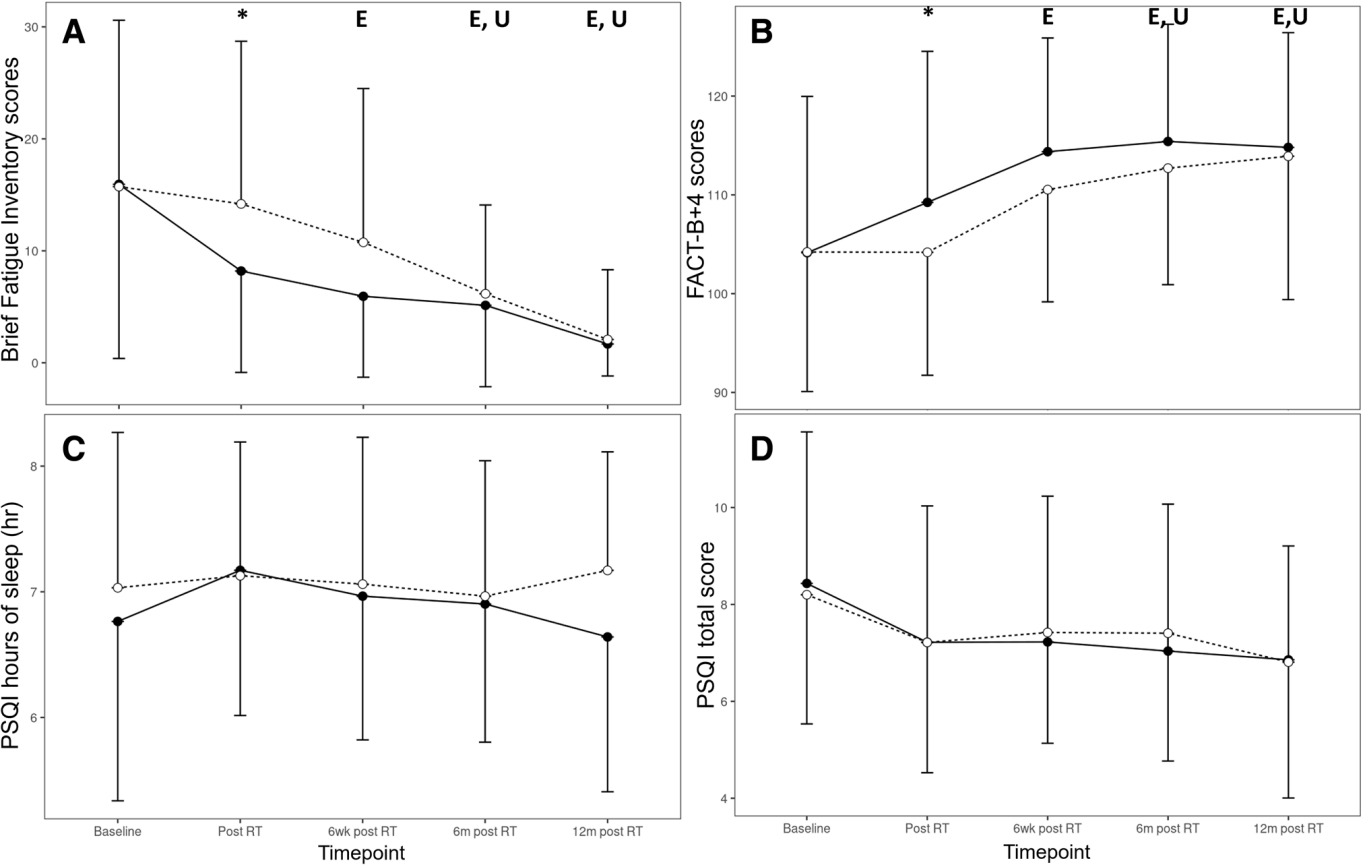
**A** Brief Fatigue Inventory, **B** FACT-B + 4, **C** Pittsburg Sleep Quality Index hours of sleep, **D** Pittsburg Sleep Quality Index total score for all time points (baseline, post-RT, and 6 weeks, 6 months, and 12-month post-RT) for both groups. Exercise: straight line and black circles; usual care: dashed line and white circles. Characters denote significant difference of either the exercise (E) or the usual care (U) groups with their respective baselines, and *denotes significant difference between groups (*p* < 0.05). Error bars denote standard deviation of the mean

For the BFI, there was a significant between-group difference post-RT, and only the exercise group experienced an improvement as early as 6 weeks post-RT compared to baseline (Fig. 5A). The usual care group improved from their baseline scores only at 6- and 12-month post-RT. At 6 weeks post-RT, being more fatigued was associated with a greater RPE during resistance exercise (*p* = 0.002) while having less fatigue was associated with a longer weekly aerobic exercise duration (*p* = 0.035).

### Quality of life

Both groups reported improved HRQoL at 6- and 12-month post-RT (Fig. 5B). However, there was a significant between-group difference post-RT (but at no other timepoint), and only the exercise group experienced an improvement as early as 6 weeks post-RT compared to baseline. Immediately post- and at 6 weeks post-RT, a poorer HRQoL was associated with a greater RPE during resistance exercise (*p* = 0.006, *p* < 0.001).

### Sleep duration and quality

There were no changes in sleep duration or total PSQI score for any group at any time point (Fig. 5C and D). Exercise dosage did not influence sleep duration at any point during the study. Immediately post- and at 6 weeks post-RT, having more trouble sleeping was associated with a greater RPE during resistance exercise (*p* = 0.019, *p* = 0.001) but RPE during aerobic exercise was associated with less sleep trouble (*p* = 0.014, *p* < 0.001). Moreover, at 6 weeks post-RT, having less trouble sleeping was associated with a greater weekly aerobic exercise duration (*p* = 0.021). The average global sleep quality score remained greater than 5 for both groups in all timepoints, indicating poor sleep quality.

### Physical activity

**Fig. 6:**
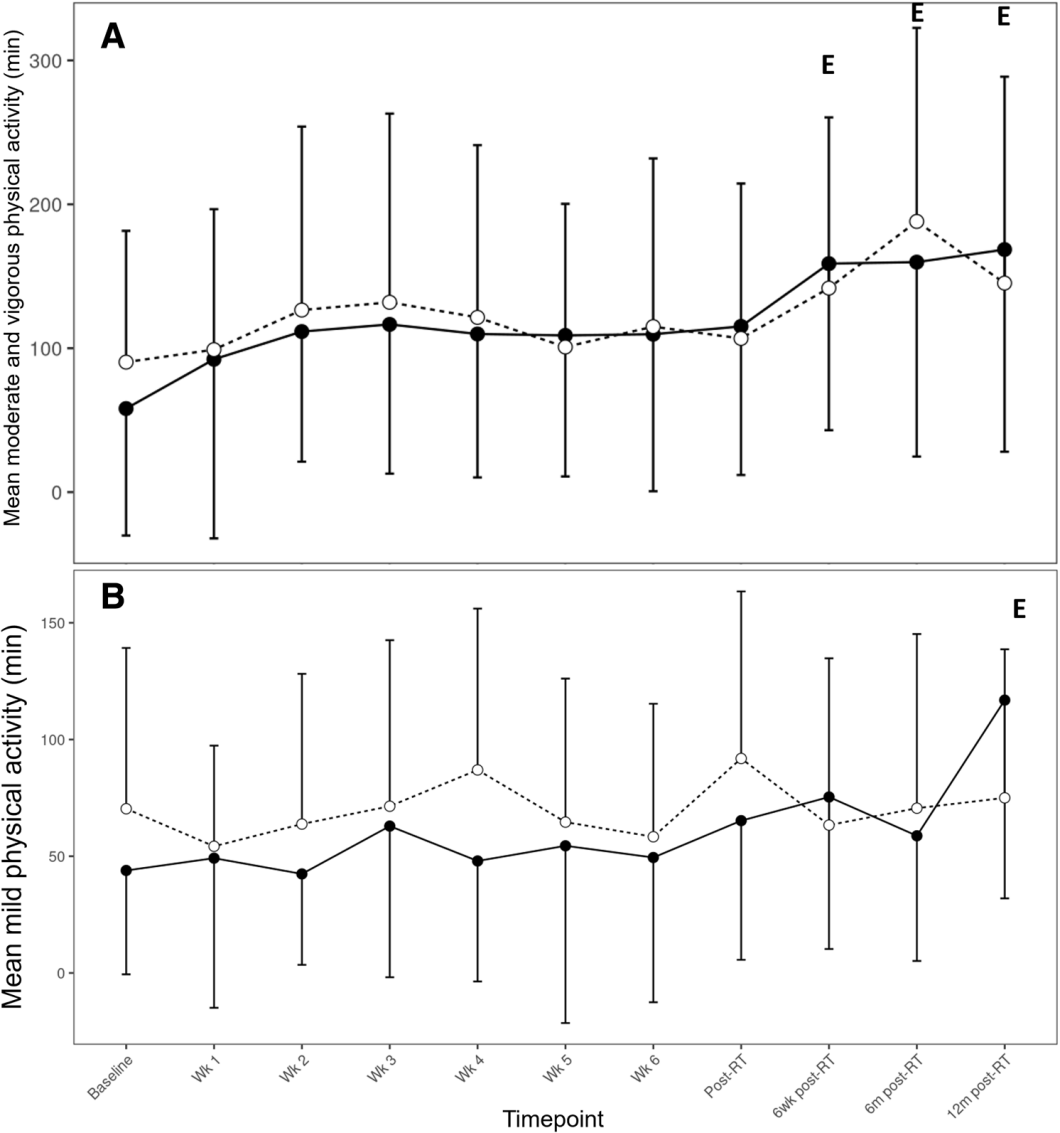
**A** Mean minutes of moderate and vigorous physical activity (sum of minutes with a double weighting on vigorous intensity minutes), **B** mean minutes of mild physical activity. Exercise: straight line and black circles; usual care: dashed line and white circles. The (E) character denotes significant difference of the exercise group with its baseline (*p* < 0.05). Error bars denote standard deviation of the mean

Compared to baseline, MVPA significantly increased for the exercise group only at 6 weeks (*p* < 0.001), 6 months (*p* < 0.001), and 12 months (*p* < 0.05) post-RT, while mild physical activity increased only for the exercise group at 12 months (*p* < 0.05) post-RT (Fig. 6). However, there were no significant differences between groups at any time point.

## Discussion

The purpose of this study was to examine the efficacy of a 12-week home-based resistance and aerobic exercise program on CRF, HRQoL, and sleep quality and duration in BCa patients during and up to 12 months after RT, and investigate how CRF, HRQoL, and sleep quality and duration affect the participant’s ability to follow their prescribed exercise program. We found that CRF was present at baseline and persisted during RT. The exercise group had a quicker reduction in CRF compared to the usual care group. Moreover, there was a significant difference in the HRQoL during RT between groups, and a quicker HRQoL improvement post-RT for the exercise group, with no changes in sleep quality or duration. Regarding the second aim, being more fatigued, poorer HRQoL, and trouble sleeping was associated with greater RPE during resistance training. Less fatigue and less trouble sleeping was associated with greater weekly aerobic exercise duration and higher RPE during aerobic exercise.

CRF symptoms have been shown to significantly increase in frequency over a typical 5-week RT course in women with BCa [3]. In our study, however, CRF was present from baseline, albeit not clinically significant [18], and persisted during the course of RT. Moreover, for the usual care group, up to 6 weeks post-RT was required for a significant improvement in CRF and it required up to 12-month post-RT to fully resolve. In addition, HRQoL significantly improved for the usual care group only at 6-month post-RT while RT did not seem to influence sleep duration or quality.

There was a significant improvement in CRF only in the exercise group during RT (with a significant difference between the groups), while the usual care group experienced a significant improvement only after 6 weeks post-RT based on the FACIT-F. Similar results were observed in a study comparing 12 weeks of progressive resistance training (performed twice per week) to a relaxation group in women with BCa starting RT, where there were significant between-group differences for self-reported CRF using the Fatigue Assessment Questionnaire, especially in regard to physical fatigue [33]. CRF decreased from baseline to post-intervention in the exercise group, but there was no change in the relaxation group. In our study however, a between-group significance difference occurred only during RT and immediately post-RT, and not at subsequent time points. Moreover, the difference between groups at weeks 5, 6 and post-RT was ≥ 4, which has been established as clinically significant [14]. Similarly, even though the BFI was not measured weekly during RT, there was a statistically significant difference between the groups immediately post-RT, but not at later time points. Although patients in the study did not experience clinically significant CRF, exercise played a role in reducing CRF, although this only occurred during the course of the exercise intervention, and not during the post-intervention period.

HRQoL was also significantly different between-group post-RT, and only the exercise group significantly improved by 6 weeks post-RT. Similar results were observed in the study of Steindorf et al. where women with BCa commencing RT underwent 12 weeks of progressive resistance training (performed twice per week) or relaxation [33]. HRQoL (EORTC QLQ-C30) improved from baseline to post-intervention only in the exercise group, but the difference between groups did not reach statistical significance. In our study, we found a significant difference only at 6 weeks into the intervention (immediately post-RT), with separation of the two groups persisting at 12 weeks (not statistically significant) before merging at 6-month post-RT, which is similar to the CRF findings. This suggests that exercise is effective only while it is being undertaken, and might also explain the lack of significant differences between groups in the study of Steindorf et al., as HRQoL was not measured during the actual intervention [33].

The average global sleep quality score remained greater than 5, indicating poor sleep quality; however, the score neither improved nor declined at any time point during the study. To the best of our knowledge, there are no previous studies examining the effects of exercise on sleep duration and quality in BCa patients undergoing RT. However, one previous study examined the effects of a standard dose (25–30 min of aerobic exercise), a higher dose (50–60 min of aerobic exercise), or a combined dose of exercise (50–60 min of aerobic and resistance exercise), performed 3 times a week on sleep quality (PSQI) in women with BCa receiving chemotherapy [7]. It was found that the improvement in the high dose group was superior to the combined group, which was superior to the standard group for sleep quality and duration. In our study, sleep quality was relatively unaffected by RT; however, the participants in the study of Courneya et al. were undergoing chemotherapy [7], which might have more debilitating effects on the quality and duration of sleep than RT, and therefore a more pronounced difference could have been realized as a result of the exercise intervention.

There was variability in the exercise dosage in our study. For example, there were a few outliers who performed 220 min of aerobic exercise per week, which could have been women who were already exercising regularly, but most women performed 30–40 min of aerobic exercise weekly. There were numerous associations of resistance and aerobic exercise dosage across multiple questionnaires (BFI, FACT-B + 4, PSQI), indicating that higher resistance exercise RPE was associated with poorer CRF, HRQoL, and sleep quality. This most likely indicates that exercise was perceived to be harder by the participants who were experiencing more CRF, and not necessarily that exercising at higher effort levels was causing more fatigue and worse HRQoL, since exercise in our study was shown to decrease CRF and improve HRQoL. It has been previously shown that higher resistance and aerobic exercise intensity can result in significantly better CRF outcomes in patients undergoing neoadjuvant therapy for BCa [11]. Based on our finding that participants with higher CRF perceive exercise to be harder, strategies such as autoregulation and periodization combined with exercise formats such as high intensity interval training and cluster sets need to be implemented, to enable patients with CRF to exercise at higher intensities to achieve for CRF reduction.

Even though most of the participants were able to complete more than one resistance session per week, most of the women were not able reach the target of 150 min of aerobic exercise per week, with few exceptions. This suggests that if the patient is experiencing CRF, their capacity for aerobic exercise volume may be compromised, which might help explain why there was such a high variation in the responses. Nevertheless, our findings are that even the much smaller dosages of exercise performed in our study can have significant effects on CRF and HRQoL during and after RT. Importantly and of interest, these dosages are less than those currently recommended in exercise guidelines for cancer patients and survivors, yet improvements are observed [29, 34, 35]. Some participants that withdrew in the exercise group cited reasons of being overburdened filling the exercise logbook and questionnaires; however, this was not due to the exercise itself, but due to the requirement to collect the data. No adverse effects of the exercise intervention were reported. Given the above, the home-based exercise intervention used in this study was safe and efficacious in reducing CRF and improving HRQoL. It should be noted that all the RT-related adverse effects occurred in participants of the exercise group. These adverse effects were skin burns, viral infection, diarrhea or vomiting and unlikely to be related to the exercise intervention, although future studies are required to confirm this.

The safety and efficacy of exercise during RT has also been demonstrated in a previous study where women took part in a 12-week resistance exercise program (2 times per week) in an exercise clinic under supervision [33]. However, in our study, the participants were given an exercise guide and low-cost exercise equipment, to perform unsupervised exercise at home, which was still effective in reducing CRF and improving HRQoL. A home-based protocol might be preferable for patients, as it is low-cost, does not require travel or supervision, and can be performed at a time and location that the participant prefers. These features may provide substantial comfort to patients suffering from high CRF and poor HRQoL.

The exercise program in this study might have induced changes in the participants’ behavior around physical activity. The exercise group had significant improvements in both ‘moderate and vigorous’ and mild physical activity up to 12 months after the end of the exercise intervention as shown in the results of the Godin questionnaire (Fig. 6), while a similar improvement was not observed in the usual care group. Thus, apart from the direct beneficial effects on reduction in CRF and improving HRQoL during RT, home-based exercise protocols might result in changes in the physical activity of participants that persist well after the end of the program.

Despite the relatively large sample size, the long follow-up period (12 months), and the comprehensive measures of CRF, HRQoL, sleep, and physical activity, this study has some limitations worthy of comment. We did not have a control group that was not undergoing RT, to allow us to better understand the effects of RT on our assessed endpoints. Due to the urgency to manage the cancer, it would have been challenging to find participants who did not undergo RT for 12 months and were at the same cancer stage. Moreover, there was already CRF at baseline, which might have been caused by other therapies such as chemotherapy and hormonal therapy. Patients with cancer undergo multiple therapies and, as such, it is challenging to isolate a sample that has not undergone previous treatments. However, not only were the groups randomized in a balanced fashion according to previous cancer therapies, but there was also no difference at baseline in CRF between groups.

In conclusion, home-based exercise during RT is safe and effective in reducing CRF and improving HRQoL. The benefits observed in this study can occur even with unsupervised home-based exercise and minimal equipment cost; however, these benefits occur when exercise is conducted regularly, and dissipate after cessation. Moreover, significant CRF and HRQoL can be achieved even with smaller dosages of exercise than what is currently recommended.
